# Use of SNPs to determine the breakpoints of complex deficiencies, facilitating gene mapping in *Caenorhabditis elegans*

**DOI:** 10.1186/1471-2156-6-28

**Published:** 2005-05-26

**Authors:** Pavan Kadandale, Brian Geldziler, Melissa Hoffmann, Andrew Singson

**Affiliations:** 1 Waksman Institute of Microbiology, Rutgers University, 190 Frelinghuysen Road, Piscataway, NJ 08854, USA

## Abstract

**Background:**

Genetic deletions or deficiencies have been used for gene mapping and discovery in various organisms, ranging from the nematode *Caenorhabditis elegans *all the way to humans. One problem with large deletions is the determination of the location of their breakpoints. This is exacerbated in the case of complex deficiencies that delete a region of the genome, while retaining some of the intervening sequence. Previous methods, using genetic complementation or cytology were hampered by low marker density and were consequently not very precise at positioning the breakpoints of complex deficiencies. The identification of increasing numbers of Single Nucleotide Polymorphisms (SNPs) has resulted in the use of these as genetic markers, and consequently in their utilization for defining the breakpoints of deletions using molecular biology methods.

**Results:**

Here, we show that SNPs can be used to help position the breakpoints of a complex deficiency in *C. elegans*. The technique uses a combination of genetic crosses and molecular biology to generate robust and highly reproducible results with strong internal controls when trying to determine the breakpoints of deficiencies. The combined use of this technique and standard genetic mapping allowed us to rapidly narrow down the region of interest in our attempts to clone a gene.

**Conclusion:**

Unlike previous methods used to locate deficiency breakpoints, our technique has the advantage of not being limited by the amount of starting material. It also incorporates internal controls to eliminate false positives and negatives. The technique can also easily be adapted for use in other organisms in which both genetic deficiencies and SNPs are available, thereby aiding gene discovery in these other models.

## Background

**Figure 1:**
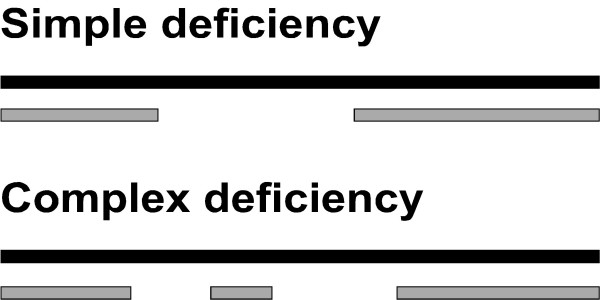
**Difference between simple and complex deficiencies. **Black bars show normal chromosomes, while grey bars represent hypothetical deficiency chromosomes. A complex deficiency deletes a large chromosomal region, but retains a small part within the larger deleted region, complicating the analysis of deficiency mapping data.

Chromosomal deletions (also called genetic deficiencies) have been used to help narrow down the location of genes in organisms ranging from *C. elegans *to human beings [1-4]. In the invertebrate model system, *Drosophila melanogaster*, deficiencies have been particularly useful for gene mapping, since the existence of polytene chromosomes and cytogenetics in this organism allows the actual visualization of the breakpoints of the deficiencies. In other organisms, however, mapping the breakpoints of deficiencies is not so straightforward. In *C. elegans*, for example, deficiencies are defined as deletion mutations that fail to complement multiple loci with the underlying assumption that the deletion would also remove the regions between these loci. Although this is generally true, deficiencies exist that are complex deletions, eliminating two distant loci, but retaining the intervening sequence (Figure 1). An example of such a complex deficiency is shown in Table 1. Genetic complementation tests [5] show that although the deficiency *sDf62 *deletes a region from map position 3.99 to 5.78 of chromosome IV, it fails to delete some genes that lie within this map interval (e.g. *unc-43 *and *lin-3*). In fact, our searches on WormBase [5] reveal that a surprising number of deficiencies in *C. elegans *are of a complex nature. When we examined a random sampling of 20 deficiencies from WormBase [5] we found that 9 of these were complex deficiencies. Although 20 is not, by any means, a large sample, it indicates that a sizeable number of deficiencies in *C. elegans *might be complex. This, coupled with the relative scarcity of usable genetic markers can confound the interpretation of deficiency data.

**Table 1:** Genetic complementation results for *sDf62*, showing it to be complex

**Position**	**Gene**	**Result**
3.99 ± 0.003	***eat-1***	+
4.58 ± 0.001	***unc-43***	-
4.66 ± 0.002	*him-8*	-
4.67 ± 0.033	let-71	-
4.81 ± 0.011	lin-3	-
4.81 ± 0.024	let-70	+
4.81 ± 0.035	*let-64*	-
4.82 ± 0.017	*sem-3*	+
4.82 ± 0.017	*let-59*	+
4.82 ± 0.033	*let-98*	+
4.82 ± 0.051	*let-100*	+
4.82 ± 0.069	*let-73*	+
4.83 ± 0.042	*dif-2*	+
4.86 ± 0.069	*let-311*	+
4.96 ± 0.069	*let-655*	+
4.97 ± 0.069	*let-91*	+
5.00 ± 0.046	***rib-1***	+
5.18 ± 0.003	***dpy-20***	+
5.43 ± 0.012	***unc-22***	+
5.51 ± 0.038	*pha-3*	+
5.73 ± 0.035	***let-68***	+
5.75 ± 0.053	***let-656***	+
5.78 ± 0.037	*let-309*	+
6.01 ± 0.016	***let-99***	-
6.13 ± 0.083	*let-97*	-
7.97 ± 0.010	***unc-30***	-

A "+" result indicates non-complementation, while a "-" indicates complementation. Loci in bold are cloned genes. Table derived from WormBase [5].

**Figure 2:**
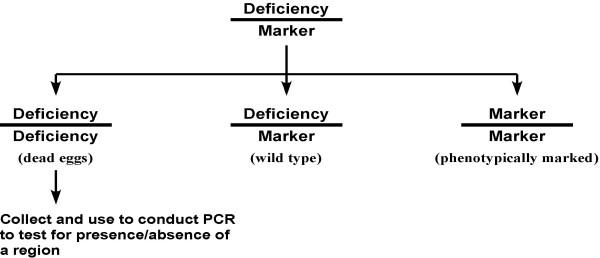
**PCR can be used to identify deletion breakpoints. **Schematic showing previous methods to identify the breakpoints of deletions using molecular biology tools. A heterozygous deficiency strain is allowed to fertilize itself. A fourth of the resulting embryos will be dead, since the homozygous deficiency is lethal. These dead eggs are collected and used for PCR to test for the presence of regions of interest.

In order to address the problem of deletion breakpoint mapping in *C. elegans*, previous work [6] has attempted to use PCR to determine the breakpoints of such deficiencies (Figure 2). Large deletions in the homozygous state are usually lethal to the developing *C. elegans *embryo. Thus, from a plate of worms heterozygous for the deletion, one can pick individual dead eggs that have been laid, assuming that these must be homozygous for the deletion. One can then use PCR to test whether a particular region of interest is present in such samples, thereby determining the extent of the deletion. This method, which relies on a negative result (i.e., lack of a PCR product), has several drawbacks: (a) The amount of starting material is restricted to a single, or few dead eggs. (b) Chitinase treatment is required to remove the egg shell. (c) False positives can result if the PCR merely fails to work properly on the limited sample material. (d) False negatives can result if an embryo dies for reasons other than that it was homozygous for the deletion.

Another method that could be used to achieve the same result is Southern Blotting [7]. However, this method is tedious, involving the preparation of high molecular weight genomic DNA, running gels and probe hybridization, and requires a larger starting amount of DNA.

In human research, taking advantage of the growing number of SNPs identified in the human genome, approaches are being developed to use SNPs to identify regions of loss of heterozygosity (LOH), and/or deletions in chromosomes [8]. The availability of a strain of *C. elegans*, CB4856, which is predicted to harbor about 1 SNP per kilobase of genomic sequence [9,10] when compared to the wild-type strain (N2), led us to believe that we could adopt similar methods for use in *C. elegans*, thereby overcoming the limitations of using deficiencies in this model system. In fact, the *C. elegans *SNP database already contains about 6,000 confirmed and predicted SNPs between the N2 and CB4856 strains [11], which corresponds to a SNP density of about 1 per 15 Kb of sequence. As the use of SNPs to map genes increases [12,13], we expect that many more SNPs will be discovered, making their use even more attractive. We report here the use of SNP mapping, in conjunction with traditional genetic deletion mapping to obtain a better map of the breakpoints of a complex deficiency, thereby greatly assisting the process of gene mapping and characterization.

## Results and Discussion

### Identification of *ozDf2 *as a complex deficiency

**Figure 3:**
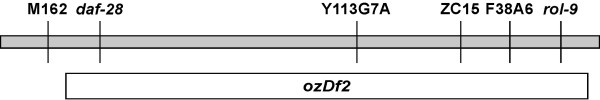
**Schematic representation of the chromosomal region around *spe-19***. The grey bar represents a normal chromosome. The relative positions of the various SNPs and loci mentioned in the text are indicated. Note that the figure is not drawn to scale. The region expected to be deleted by the deletion *ozDf2 *is indicated by the white box labelled "*ozDf2*."

**Figure 4:**
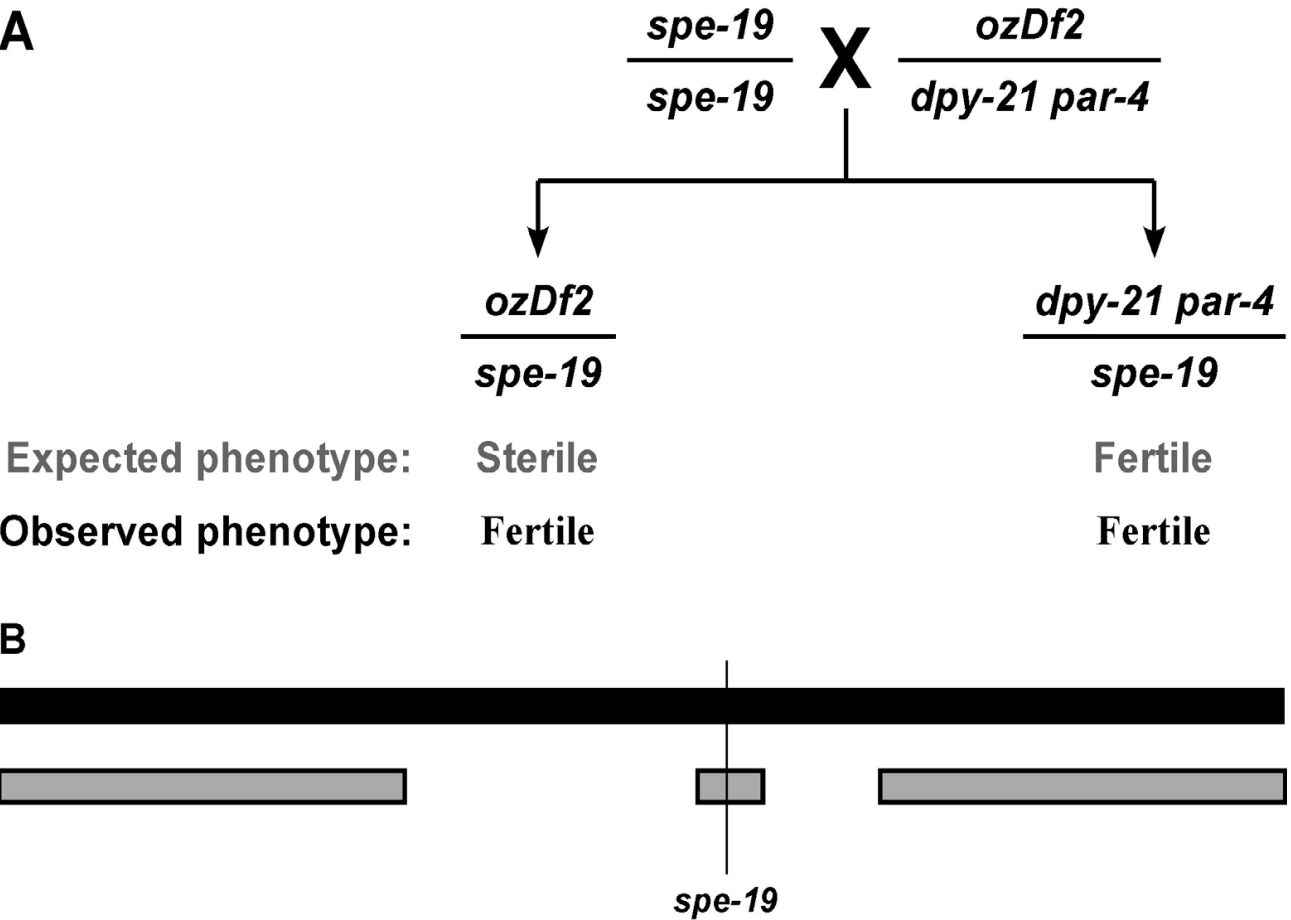
**Genetic complementation test of *ozDf2*. **(A) *ozDf2 *was tested by a standard genetic complementation test to check whether it uncovers *spe-19*. The *ozDf2*/*spe-19 *worms were expected to be sterile, but were fertile. Thus, the deletion complements *spe-19 *although it is predicted to delete *spe-19*. (B) This implies that *ozDf2 *must be a complex deficiency, retaining the *spe-19 *containing region, within a deletion that surrounds this locus.

We encountered a complex deficiency while trying to map the gene *spe-19 *in *C. elegans*. Initial mapping put the gene within the interval deleted by the large deficiency, *ozDf1 *(spanning the region from +13.18 to +25.11 of chromosome V). Standard SNP mapping analysis [12] placed *spe-19 *within the region bounded by SNP M162 (map position +23) on the left and SNP ZC15 (map position +25) on the right (BG, manuscript in review). According to data from WormBase [5], this region is expected to be deleted (Figure 3) by the smaller deficiency, *ozDf2 *(which deletes the region from +23.5 to +25.11 of chromosome V by genetic complementation tests). When we performed the genetic complementation test, we found, to our surprise, that *ozDf2*/*spe-19 *animals were fertile suggesting that *ozDf2 *does not delete *spe-19*. We therefore hypothesized that *ozDf2 *was a complex deficiency (Figure 4).

### Use of SNPs to map the breakpoints of *ozDf2*

**Figure 5:**
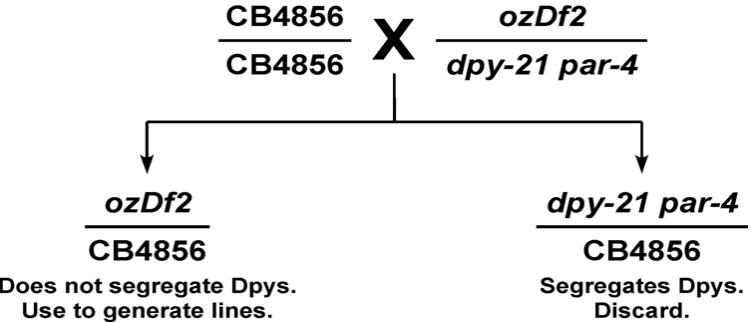
**Generation of lines for SNP mapping of deletion breakpoints**. The cross performed to obtain the lines used for the SNP mapping of the deficiency breakpoints is outlined. The details of the crosses are described in the Methods.

**Figure 6:**
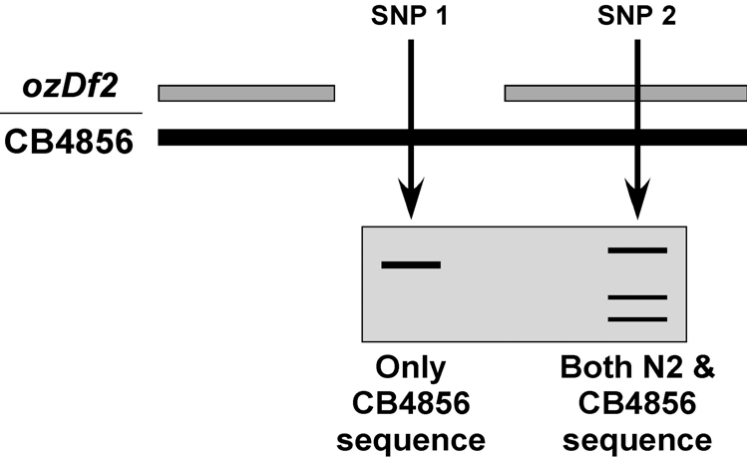
**Schematic representation of the results expected for the SNP analysis**. Schematic showing what banding patterns would be expected in SNPs that are deleted by the *ozDf2 *deficiency (hypothetical SNP 1) and in SNPs that are not (hypothetical SNP 2). The boxed region shows the hypothetical banding pattern expected on an agarose gel for the two SNPs. Note that SNPs are chosen so that for a given SNP, the wild-type (N2) sequence is digested by an appropriate restriction enzyme, whereas the CB4856 SNP sequence is not digested by the same enzyme.

**Figure 7:**
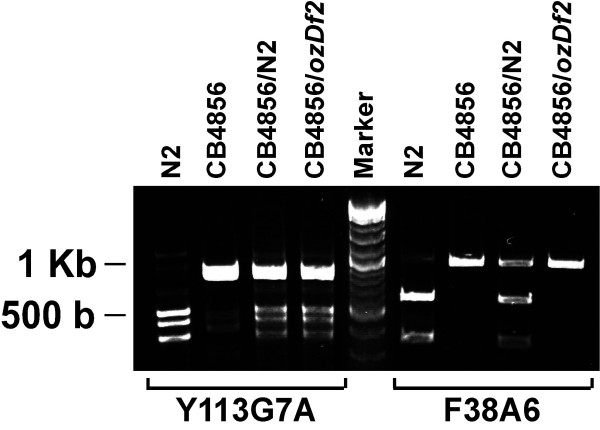
**Representative results of the SNP analysis. **Representative results of SNP mapping of *ozDf2*'s breakpoints. Lanes are labeled with the genotype of the strain used. Marker used was New England Biolabs 2-Log ladder. Note that SNP F38A6:14347 is deleted in *ozDf2 *(no N2 bands in *ozDf2*/CB4856), whereas SNP Y113G7A:29658 is not deleted (N2 bands seen in *ozDf2*/CB4856).

In order to test whether *ozDf2 *was, indeed, a complex deficiency, and to map its breakpoints, we used a series of SNPs in the region [11] to obtain a higher resolution map of the deficiency. For this purpose, we used SNPs whose sequence change results in the loss of a restriction enzyme recognition site in the CB4856 animals. Thus, if we see any product that is digested, even partially, we can confirm the presence of the wild-type (strain N2) sequence. We generated the appropriate lines (see Methods and Figure 5) to use SNPs to map the deficiency breakpoints. We obtained animals that are heterozygous for the deficiency *ozDf2*, the homologous chromosome being from strain CB4856. Thus, in these animals, in chromosomal regions lost by the deletion *ozDf2*, we should see sequences corresponding only to the CB4856 SNP sequence. In regions not deleted by *ozDf2*, we expect both the wild-type (strain N2) sequence as well as CB4856 sequence (Figure 6). We show here the results from two SNPs – F38A6:14347 and Y113G7A:29658 – both of which are predicted to be deleted by *ozDf2 *(Figure 7). As expected, the heterozygous *ozDf2*/CB4856 animals showed only the uncut CB4856 band for SNP F38A6:14347. However, SNP Y113G7A:29658 shows both the uncut CB4856 band as well as the cut N2 bands, indicating that at this region, the N2 sequence was present (Figure 7). Since we expected the N2 sequence of Y113G7A:29658 to be absent in the *ozDf2*/CB4856 worms (the *ozDf2 *deletion is expected to also have lost the Y113G7A:29658 region – see Figure 3), the retention of N2 sequence around Y113G7A:29658 confirms that *ozDf2 *is a complex deficiency. Thus, *ozDf2 *deletes the region from +23.5 to +25.11 of chromosome V, but retains a part of this interval, around the SNP Y113G7A:29658. This result indicates that *spe-19 *must localize to the region around SNP Y113G7A:29658, since this area is retained in the complementing deficiency, *ozDf2*.

We have shown here that using SNPs as genetic markers in model systems greatly eases the problems of defining the breakpoints of deficiencies, both simple and complex. This allows us to use them as powerful tools in narrowing down the region of interest when cloning genes.

## Conclusion

Our use of a combination of genetic complementation data along with SNP mapping of deficiency breakpoints has allowed us to rapidly narrow down the region of interest for cloning *spe-19*. Deficiency mapping in a number of organisms, including *C. elegans*, has been beset by the lack of a convenient method to correlate genetic complementation data with the physical map, confounding our ability to accurately define the breakpoints of deficiencies. Previous methods used to overcome this obstacle depended on a negative result [6,7], which is often untrustworthy. Using SNPs, not only is the correlation between the genetic and physical maps easily achieved, but we can also, potentially, generate higher resolution maps of the breakpoints of both simple, as well as complex deficiencies. Additionally, the amplification of the identical locus from the CB4856 chromosome serves as a good internal control, eliminating false positives and false negatives and allowing us to interpret our data with a very high degree of confidence. The fact that we can use heterozygous animals, without the need to generate homozygous, dead embryos overcomes the limitations of previous methods which relied exclusively on negative results, such as using PCR to detect the presence or absence of a sequence. We chose to use bulk worm lysates from a heterozygous line as our source of DNA. However, the technique would work just as well if single worms (after confirming their genotype) were used as the source of DNA. We believe that using lines has the following advantages:

1. The line can be frozen away and then rethawed at a later date if further analysis is required.

2. Multiple SNPs can be tested using the same lysate, as opposed to requiring multiple, single worm lysates.

3. Lysates can be prepared in bulk, in replicates, for independent and robust confirmation of all results obtained by this method.

4. No special care needs to be taken for preparation of DNA for SNP detection. DNA is made from the lines using a simple, scaled up version of the method used to make worm lysates for single worm PCR.

We initially chose to use SNPs that caused an abrogation of a restriction enzyme recognition site in the CB4856 sequence, thereby eliminating any artefacts due to incomplete restriction enzyme digestion. However, by running a control reaction to ensure that enzyme digestion has progressed to completion, one could also use SNPs that cause the loss of a restriction enzyme recognition site in the N2 sequence. Additionally, this method allows the use of SNPs that are only different in sequence, causing no changes in restriction enzyme recognition sites. A simple sequencing reaction can be done to check whether the *Deficiency*/CB4856 worms had only CB4856 sequence (which would be the case if the deficiency deletes the region of the SNP), or whether both N2 and CB4856 sequence was present (indicating that the SNP region is not lost in the deletion being tested). In addition to the CB4856 strain which we have used, other wild isolates of *C. elegans *which harbour SNPs [9] can also be used, expanding the possibilities of this technique.

While we have shown this method to work in the model system *C. elegans*, a similar approach can be applied in any organism where deficiencies/deletions exist and where SNP mapping is possible.

## Methods

### Culturing of *C. elegans *strains

Worms were grown and cultured according to standard protocols described elsewhere [14]. The strains used for this work were: *spe-19*, *ozDf2*/*dpy-21 par-4*, N2 (the wild-type) and CB4856.

### Survey of deficiencies on WormBase

We looked at the complementation data for 20 deficiencies chosen at random. We ensured that deficiencies from all chromosomes were included in the analysis and that the deficiencies chosen had their left and right boundaries defined by cloned genes (this allowed us to set the other boundaries of the deficiency with certainty). We then looked at the complementation data and scored the deficiency as "complex" if at least one of the genes that were within the outer boundaries of the deficiency was complemented by the deficiency. While scoring the complex deficiencies, we made sure that the internal locus that was complemented was either cloned or had multiple, corroborating mapping data. If the deficiency failed to complement all the loci that were within the outer boundaries, it was scored as a simple deficiency. The deficiencies that we scored as simple are: *nDf20*, *sDf10*, *mnDf16*, *nDf3*, *tDf1*, *mnDf1*, *ccDf1*,*eDf2*, *tDf5*, *ctDf2 *and *ctDf3*. The complex deficiencies we found are: *hDf10*, *hDf17*, *nDf19*, *nDf9*, *eDf18*, *qDf8*, *eDf3*, *maDf4 *and *nDf27*.

### Genetic complementation test of *ozDf2*

Since *spe-19 *males are fertile, we mated *ozDf2*/*dpy-21 par-4 *with *spe-19 *males. Of the outcross F_1 _progeny, we would expect half to be of the genotype *ozDf2*/*spe-19 *and consequently, sterile. The other half would be fertile, *spe-19*/*dpy-21 par-4 *worms. However, all the F_1 _progeny were fertile, indicating that the deletion *ozDf2 *complemented *spe-19*, belying our expectations. The presence of the deletion and of *spe-19 *was confirmed by looking at the F_2 _progeny. Half the F_1 _progeny segregated wild-type animals, dead eggs and sterile worms, indicating that their genotype must be *ozDf2*/*spe-19*. Thus, *ozDf2 *complements *spe-19*.

### Generation of lines for SNP mapping of the breakpoints of *ozDf2*

We crossed *ozDf2*/*dpy-21 par-4 *hermaphrodites to CB4856 males. These *ozDf2*/*dpy-21 par-4 *heterozygotes are wild-type, but *ozDf2 *homozygous animals are embryonic lethal and *dpy-21 par-4 *homozygotes are short and fat – a phenotype referred to as Dumpy, or Dpy. F_1 _animals that segregated Dpy progeny were discarded (these would have been *dpy-21 par-4*/CB4856), and only the remaining, heterozygous animals (which are all *ozDf2*/CB4856) were used to set up lines that were further analyzed for their SNP sequences (Figure 5). Individual *ozDf2*/CB4856 worms were picked to separate culture plates. These worms are fertile and generate independent lines which were used for further analysis. Each line was generated within 6 days of picking the founder worm, ensuring that the N2 deficiency chromosome would not be underrepresented significantly in the line. Thus, each individual line would contain a mixture of CB4856 and *ozDf2 *chromosomes, which provide abundant material for the analysis of multiple SNP sequences. We established three independent heterozygous lines for analysis.

### SNP mapping of the breakpoints of *ozDf2*

Crude genomic DNA was prepared from the three *ozDf2*/CB4856 lines by methods described previously [15]. We used the Vector NTI suite to design primers that amplified ~500 bp upstream and downstream of the SNP, resulting in a PCR product that was approximately 1 Kb. The details of the primers used and the PCR products generated are as follows:

SNP Y113G7A:29 – The expected product size is 1033 bp using the forward primer (GTAGGAAGTGGTGCCAGATGACGAGG) and the reverse primer (GTCTCAATTTGTGAGCGCATGTC).

SNP F38A6:14347 – The expected product size is 961 bp using the forward primer (TGGTTCCCCTCTACCAGATGCC) and the reverse primer (CCAACACAACTCAATCTAATGTTTACC).

We used these primers to amplify the corresponding product from each of the three genomic DNA preparations of the *ozDf2*/CB4856 lines using the following PCR conditions: denature at 94°C for 30 seconds, anneal at 60°C for one minute, extend at 72°C for 1 minute and repeat for 30 cycles. The PCR product was then digested with an appropriate restriction enzyme, and the products analysed on a 1.5% agarose gel run using the SB buffer. For SNP Y113G7A:29, *Xmn*I was used to digest the PCR product and for SNP F38A6:14347, *Dra*I was used.

## Authors' contributions

PK conceived of using SNPs to analyse deletion breakpoints, carried out the SNP analysis and drafted the manuscript. All of the genetic complementation mapping was carried out by BG. MH aided with the molecular biology. AS participated in designing the experiments and in their coordination and helped to draft the manuscript. All work was conducted in the laboratory of AS.
